# Cardiovascular Dysautonomia in Patients with Parkinson’s Disease and Hypertension: A Cross-Sectional Pilot Study

**DOI:** 10.3390/jcm14072225

**Published:** 2025-03-25

**Authors:** Delia Tulbă, Aida Cristina Tănăsoiu, Ana-Maria Constantinescu, Natalia Blidaru, Adrian Buzea, Cristian Băicuș, Laura Dumitrescu, Eugenia Irene Davidescu, Bogdan Ovidiu Popescu

**Affiliations:** 1 Department of Clinical Neurosciences, “Carol Davila” University of Medicine and Pharmacy, 050474 Bucharest, Romania; delia.tulba@umfcd.ro (D.T.); natalia-ioana.dima@drd.umfcd.ro (N.B.); laura.dumitrescu@drd.umfcd.ro (L.D.); eugenia.davidescu@umfcd.ro (E.I.D.); 2 Department of Neurology, Colentina Clinical Hospital, 020125 Bucharest, Romania; aida-cristina.tanasoiu@rez.umfcd.ro (A.C.T.); ana-maria.constantinescu@rez.umfcd.ro (A.-M.C.); 3 Department of Cardio-Thoracic Pathology, “Carol Davila” University of Medicine and Pharmacy, 050474 Bucharest, Romania; catalin.buzea@umfcd.ro; 4 Department of Cardiology, Colentina Clinical Hospital, 020125 Bucharest, Romania; 5 Colentina-Research and Development Center, Colentina Clinical Hospital, 020125 Bucharest, Romania; cristian.baicus@umfcd.ro; 6 Department of Internal Medicine, “Carol Davila” University of Medicine and Pharmacy, 050474 Bucharest, Romania; 7 Department of Internal Medicine, Colentina Clinical Hospital, 020125 Bucharest, Romania; 8 Laboratory of Cell Biology, Neurosciences and Experimental Myology, “Victor Babeș” National Institute of Pathology, 050096 Bucharest, Romania

**Keywords:** cardiovascular autonomic dysfunction, orthostatic hypotension, non-dipping

## Abstract

**Background/Objectives**: Parkinson’s disease (PD) and hypertension are often coexistent conditions that interact in entwined ways at various levels. Cardiovascular autonomic dysfunction (CAD), a non-motor feature of PD occurring across all stages, alters blood pressure (BP) regulation. **Methods**: We conducted a cross-sectional study enrolling patients with PD and primary hypertension, without diabetes mellitus or other causes of secondary CAD, aiming to characterize BP profiles/patterns by ambulatory BP monitoring. We also sought associations between different CAD phenotypes and PD characteristics, disability, and cardiovascular comorbidities. **Results**: We included 47 patients with a median age of 71 years, PD duration of 9 years, and Movement Disorder Society-sponsored revision of the Unified Parkinson’s Disease Rating Scale (MDS-UPDRS) Part III score of 40. Diurnal and nocturnal BP values were within the reference range, but BP load was excessive. Almost one-third had neurogenic orthostatic hypotension (OH) and 80% were non-dippers. The overall burden of non-motor symptoms was significant in these phenotypes. Patients with neurogenic OH were more prone to constipation, anxiety, and urinary problems, whereas gustatory dysfunction, loss of libido, and erectile dysfunction were more frequently reported by non-dippers. No significant differences with regard to cognitive decline were identified in subjects with and without neurogenic OH. Neurogenic OH was symptomatic in 78% of the cases, whereas 56% of those with orthostatic symptoms did not have OH at repeated measurements. **Conclusions**: Neurogenic OH is an independent predictor of disability in patients with PD and hypertension, after adjusting for PD duration, Hoehn and Yahr stage, levodopa equivalent daily dose (LEDD), and Montreal Cognitive Assessment (MoCA) score.

## 1. Introduction

Parkinson’s disease (PD) and hypertension are two prevalent conditions that often coexist, particularly in aging populations. While hypertension is a leading risk factor for cardiovascular diseases, PD is a neurodegenerative disorder primarily affecting motor function due to progressive dopaminergic neuronal loss. Apart from the core clinical motor syndrome (parkinsonism), many non-motor features occur in PD as a result of non-dopaminergic neural structures involvement [1,2,3]. Dysregulation/failure of the sympathetic noradrenergic system and parasympathetic cholinergic system (baroreflex failure) results in cardiovascular autonomic dysfunction (CAD) [4,5], which is part of the spectrum of non-motor manifestations in PD and occurs across all disease stages, including prodromal [6]. Blood pressure (BP) regulation is often altered in PD due to CAD [7,8], leading to BP abnormalities such as orthostatic hypotension (OH), supine hypertension (SH), nocturnal hypertension (NH), and postprandial hypotension (PH), which are frequently observed in PD patients, irrespective of vasoactive treatment [9]. Moreover, different manifestations of CAD (as well as other types of dysautonomia) could assist the early differential diagnosis in synucleinopathic and tauopathic parkinsonisms and their subtypes [10,11,12]. For instance, the absence of any of the common nonmotor features (including CAD manifestations such as symptomatic orthostasis) despite 5 years of disease duration is a red flag for PD, but so is the presence of severe autonomic failure (including neurogenic OH with an orthostatic BP decrease by at least 30/15 mmHg within 3 min of standing) [13].

Another possible contributor to CAD in PD is the disrupted orexinergic system, which normally displays extensive projections to and from various cardiovascular brainstem nuclei, such as the paraventricular nucleus, nucleus tractus solitarius, and rostral ventrolateral medulla [14,15,16,17]. Orexin receptors and hypochretin-1/orexin-A (OXA) are also expressed in peripheral tissues pertaining to the autonomic nervous system (e.g., adrenal glands) [18], additionally highlighting their potential role in CAD. In postmortem studies on PD patients, a reduction in hypothalamic orexin neurons progressing with disease stage was noticed [19,20]. Moreover, a recent meta-analysis concluded that PD patients have lower cerebrospinal fluid OXA concentrations compared with controls, but still within the normal range [21]. In another study, Huang S et al. detected significantly higher plasma OXA levels in PD patients compared with healthy controls; in the subgroup analysis, this trend remained consistent for early and medium-stage PD and was reversed for advanced-stage PD [22]. Significant correlations between plasma OXA levels and various non-motor manifestations (i.e., insomnia, rapid eye movement sleep behaviour disorder (RBD), anxiety, cognitive decline, and bladder dysfunction) were found [15,22], but CAD did not reach statistical significance, yet it was subjectively assessed (*p* = 0.360) [22].

The relationship between PD and hypertension is complex and elusive, with bidirectional interactions at various levels [23]. Hypertension has been found to be either directly proportional [24], inversely proportional [25,26,27], or not associated [28,29] with PD risk. It supposedly increases mortality in PD patients through several mechanisms—apart from promoting cardiovascular disease, which is the most frequent cause of death in these patients [30], hypertension also complicates CAD manifestations, such as OH and SH [31]. Antihypertensive treatment worsens OH [32], which is responsible for falls (with subsequent head trauma and bone fractures), cognitive decline, cardiovascular events, and physical deconditioning [6], whereas pressor agents used in OH aggravate hypertension, SH, and NH, possibly eliciting end-organ damage [33,34]. Another aspect of the PD–hypertension interconnection concerns the potential neuroprotective and/or disease-modifying effect of antihypertensive drugs [35]. Interestingly, in patients with PD, hypertension is significantly less frequent than in controls [36], and the prevalence of hypertension decreases with PD duration [31,37]. Conversely, persistent hypertension in PD patients may contribute to cerebrovascular pathology, potentially worsening neurodegeneration and cognitive decline [38].

In patients with PD, the superimposition of CAD over hypertension determines abnormal BP patterns and high circadian rhythm variability that are difficult to identify and quantify by regular BP office measurements [23]. Mistaking hypertension for SH or missing hypertension in the setting of OH could lead to wrong diagnostic assumptions and therapeutic decisions [39]. Moreover, in advanced PD, the fluctuating motor and non-motor symptoms throughout the day could deeply influence BP profiles. Ambulatory blood pressure monitoring (ABPM) is currently viewed as the method of choice for elevated BP and hypertension assessment and treatment [40]. As compared with office BP monitoring, ABPM identifies BP variations throughout the day, in relation to physical and emotional activities and stressors, therefore predicting cardiovascular events more accurately [40]. Apart from exposing white-coat hypertension and masked hypertension, ABPM also identifies BP variations over the day, in relation to daytime activities, physical and emotional stressors [40]. In PD patients, ABPM improves the detection of hypertension by 15% and characterizes the nocturnal BP profiles that tend to be abnormal in these subjects [31].

Considering the lack of data regarding BP profiles and patterns in hypertensive PD patients with CAD, we aimed to thoroughly analyze them, with a focus on circadian BP variability, OH prevalence and manifestations, and the impact of vasoactive therapies. By employing ABPM and the orthostatic BP test, we searched for distinct BP phenotypes that could guide more personalized therapeutic approaches in these patients. Our findings may have significant implications for managing hypertension in PD, ultimately improving both cardiovascular and neurological outcomes.

## 2. Materials and Methods

### 2.1. Hypothesis and Objectives

Considering that PD and hypertension are often comorbid and patients with PD frequently develop CAD, we aimed to explore this triangular relationship. We hypothesized that in patients with sporadic PD and primary hypertension, without diabetes mellitus or other secondary causes of autonomic failure, CAD (related to PD) is associated with significant disability (primary outcome). Moreover, we sought associations between CAD and PD characteristics (including non-motor phenotypes), as well as cardiovascular comorbidities (secondary outcome).

### 2.2. Study Design

We conducted a unicentric cross-sectional study enrolling patients with PD and hypertension. The inclusion and exclusion criteria are listed in Table 1 and Table 2, respectively. Adult patients with both sporadic PD and primary hypertension diagnoses admitted to the Neurology Department of Colentina Clinical Hospital in Bucharest, Romania between 1 April 2023 and 30 November 2024 who fulfilled the inclusion criteria, presented no exclusion criteria, and consented to this study were consecutively included. This study was approved by the Local Ethics Committee (No. 7/02.03.2023).

### 2.3. Patient Assessment

Apart from a comprehensive medical history review and neurological examination, clinical scales for motor and non-motor status and profiles as well as disability rating scales were assessed. Each patient completed a diary of activities and sleep as well as motor fluctuations and was subjected to a standard orthostatic BP test. The standing test consisted of at least 5 min of rest in supine position, followed by 5 min of active standing, with measurements of BP at 1, 3 and 5 min.

Blood tests and urinalysis (to exclude alternative causes of CAD), carotid/vertebral ultrasound and transcranial Doppler (to evaluate atherosclerosis), electrodiagnostic testing including nerve conduction studies +/− needle electromyography (to detect and characterize peripheral neuropathy), standard electrocardiogram (ECG), 24 h ECG Holter (for time-domain measurements of heart rate variability (HRV)), and 24 h ABPM (to detect BP profiles and patterns) were performed in each patient.

We used a combined 24 h ECG Holter and ABPM system (EC-3H/ABP, LABTECH LTD., Debrecen Hungary, Cardiospy Software, v6.1). The frequency of BP measurements differed according to the period of the day (a fixed-time method for defining daytime and nighttime periods was previously employed): at 30 min and 1 h intervals, respectively. As stated by the International Consensus, the ABPM was considered valid if the 24 h recording had ≥70% of expected measurements, 20 valid awake, and 7 valid asleep measurements [43,44].

### 2.4. Variables

For each patient, the following information was obtained by anamnesis, medical records, and paraclinical workup (listed extensively in Table S1. Patient’s medical chart):

- Demographics and socio-cultural context;
- Comorbidities (that pose cardiovascular risk);
- Hypertension stages;
- Vasoactive drugs (e.g., antihypertensives, antihypotensives, antidepressants, antipsychotics, etc.);
- PD characteristics (including disease duration from motor onset, Hoehn and Yahr stage [45], motor and non-motor phenotypes, levodopa induced motor complications, dopaminergic therapy, and device-aided therapy);
- Clinical scales: Movement Disorder Society-sponsored revision of the Unified Parkinson’s Disease Rating Scale (MDS-UPDRS) Part III [46], Non-Motor Symptoms Scale for Parkinson’s Disease (NMSS) [47], Non-Motor Symptoms Questionnaire (NMSQ) [48], Composite Autonomic Symptom Score-31 (COMPASS 31) [49], Scales for Outcomes in Parkinson’s Disease-Autonomic Dysfunction (SCOPA-AUT) [50], Lawton Instrumental Activities of Daily Living (IADL) [51], Activities of Daily Living (ADL) [52], Barthel Index for Activities of Daily Living (Barthel Index) [53], Modified Rankin scale (mRS) [54], and Montreal Cognitive Assessment (MoCA) [55];
- Blood and urine tests;
- Doppler ultrasonography findings;
- Electrophysiological parameters;
- Neurogenic OH, SH;
- Cardiac rhythm and time-domain indices of HRV;
- ABPM measurements: dipper profile, morning surge, hyperbaric index, average diurnal and nocturnal systolic BP (SBP), and diastolic BP (DBP).

### 2.5. Definitions

Patients were segregated into two groups, defined by the presence or absence of CAD (neurogenic OH and non-dipping profile) [56].

We defined neurogenic OH as a decline in SBP of ≥20 mmHg and/or DBP of ≥10 mmHg with an increase in heart rate of <15 bpm by 3 min of standing up [57,58]. In patients with SH (i.e., supine BP ≥ 140/90 mmHg), a BP decrease ≥ 30 mmHg/15 mmHg was necessary in order to establish the diagnosis of neurogenic OH [58].

A nocturnal decline in BP between 10% and 20% was considered normal dipping, whereas an exaggerated decrease (i.e., >20%) was labeled extreme dipping [59]. The non-dipping BP pattern included reduced dipping (a nocturnal decrease in BP < 10% of daytime BP level) and reverse dipping (nocturnal BP rising above the awake levels) [59].

The levodopa equivalent daily dose (LEDD) was calculated according to Tomlinson et al. [60].

### 2.6. Data Analysis

Database design and data analysis were performed using IBM SPSS Statistics 26. Categorical (nominal) variables were reported as counts and frequencies (valid percent), whereas continuous variables (ordinal and numerical) were reported as median (minimum, maximum). The normality of distribution was assessed using the Kolmogorov–Smirnov test. Considering the non-normal distribution of data, non-parametric tests (i.e., Chi-Square test and Mann–Whitney U test) were subsequently applied. Appropriate non-parametric correlation coefficients were used to determine the strength of the relationship between two variables according to their type (e.g., Kendall’s Tau for continuous–continuous variables). In the multivariate analysis (logistic regression), we adjusted for the variables that were associated with disability in the univariate analysis (i.e., neurogenic OH, Hoehn and Yahr stage, LEDD, MoCA score, and PD duration). They were selected as covariates by the “enter” method, with disability measured on the mRS (good outcome, no assistance: mRS grade 0–2; and bad outcome, assistance needed: mRS grade 3–6) as a dependent variable. Hypothesis testing was two-tailed and statistical significance was defined as *p* < 0.05.

We report missing values in the following (relevant) variables: neurogenic OH (we did not perform the orthostatic BP test in 3 patients due to the lack of cooperation, so the following analysis includes only 44 subjects with BP and hypertension) and ABPM contents (in 1 patient, the measurements were not valid). In the categorical variables with missing values, we reported the valid percentage.

## 3. Results

We included 47 patients with sporadic PD and primary hypertension, without diabetes mellitus or other causes of secondary autonomic failure. The mean age was 68.51 ± 7.86 years (48–83) and the majority were men (57.4%). Most of them lived in urban areas (78.7%) and were retired (93.6%). All subjects denied regular alcohol consumption and only one patient (2.1%) was smoking at that time.

### 3.1. Cardiovascular Comorbidities and Vasoactive Drugs

Cardiovascular and cerebrovascular comorbidities, as well as detailed intake of cardioactive and vasoactive drugs, are listed in Table 3. Apart from the antihypertensive drugs, we also recorded therapy with sympathomimetics (e.g., α1-agonists such as midodrine, β2-agonists such as bronchodilators, and β3-agonists such as mirabegron), sympatholytics (e.g., β-blockers such as propranolol and α-blockers such as tamsulosin), parasympathomimetics (e.g., cholinesterase inhibitors such as galantamine, donepezil, rivastigmine, neostigmine, and pyridostigmine), parasympatholytics (e.g., antimuscarinics such as trihexyphenidyl, trospium, oxybutynin, and solifenacin), and psychiatric medications that could induce hemodynamic changes. Dopaminergic therapy is separately discussed in the following section.

Almost half of the patients (46.8%) had a history of grade 3 hypertension. Beta-blockers and ACEIs were the vasoactive drugs most commonly prescribed (53.2% and 36.2%, respectively), followed by thiazide diuretics and dihydropyridine CCBs (23.4% and 23.4%, respectively). Interestingly, although the patients on midodrine had a documented history of moderate or severe hypertension (two patients with grade 2 hypertension and one patient with grade 3 hypertension), none of them required antihypertensive medication anymore; however, two of them had a β-blocker (bisoprolol in both cases) prescribed for either ischemic heart disease or sinus tachycardia. One of the patients also required fludrocortisone, considering that OH was not controlled with 22.5 mg of midodrine. Carotid and/or vertebral atherosclerosis diagnosed by Doppler ultrasonography was very frequent among our patients (75%). Although cardiovascular and cerebrovascular events were relatively rare within the studied group (8.5% and 8.5%, respectively), the occurrence of hypertension-related target organ damage (i.e., carotid atheroma) suggests an increased cardiovascular risk.

Interestingly, the anticholinergics were rarely prescribed in this group of patients; 4.25% of the them were taking trihexyphenidyl and 2.12% were on benztropine. No antimuscarinics for overactive bladder were used.

### 3.2. Parkinson’s Disease Characteristics

Data regarding PD course, severity, phenotype (both motor and non-motor), and therapy are recorded in Table 4.

In the studied group, the median duration of the disease was 9 years, ranging from recent motor onset to 30 years of motor disease course. Regarding PD staging, according to the Hoehn and Yahr Scale, most of the patients had bilateral motor involvement without balance problems (36.2%), whereas 29.8% had some postural instability but were physically independent. The median MDS-UPDRS Part III score was 40; although it was assessed during the off-state after an acute levodopa withdrawal of 15 h, in the majority of cases the patients did not rate that state as being their worse off, so an underestimation of motor signs could be expected.

In our patients, motor fluctuations were almost as common as peak-dose dyskinesia (47.7% and 51.1%, respectively); wearing off and delayed on were the most prevalent motor fluctuations (47.7% and 34.1%, respectively). A quarter of the patients (25%) complained of morning and/or night akinesia, whereas complex motor fluctuations were infrequent (unpredictable off in 9.3% of the cases and no on in 11.4% of the cases).

The median MoCA score was 24, which would correspond to a mild cognitive impairment (MCI) (https://mocacognition.com/, accessed on 16 December 2024). Indeed, 55.3% of the patients were classified as having MCI based on their (or their informants’) assertion that cognitive decline did not significantly interfere with their ability to perform routine activities or in functional areas; 42.6% were normocognitive; and 2.1% had major neurocognitive disorder according to the International Classification of Diseases, Eleventh Revision (ICD-11), World Health Organization (WHO) 2019/2021. We did not label the neurocognitive disorder PD-MCI or PD dementia (PDD) due to insufficient data to make this assumption (e.g., brain imaging). Other non-motor symptoms were also very frequent: almost two-thirds of our patients reported constipation (67.4%), orthostatic dizziness (65.2%), urinary urgency (63%), and sleep onset and/or maintenance insomnia (61.7%).

The majority of the patients were treated with oral levodopa (91.5%) and almost one-quarter (23.4%) used controlled-release formulations. Dopamine agonists were prescribed in almost 60% of the patients, MAO-B inhibitors in 45%, and COMT inhibitors in 35%. A quarter (*n* = 12; 25.5%) had a device-aided therapy, namely continuous subcutaneous apomorphine infusion (*n* = 5; 10.6%), levodopa–carbidopa–entacapone intestinal gel infusion (*n* = 5; 10.6%), deep brain stimulation of globus pallidus interna (*n* = 1; 2.1%), and continuous subcutaneous foslevodopa/foscarbidopa infusion (*n* = 1; 2.1%).

One-third of the patients (34.9%) had axonal polyneuropathy detected with nerve conduction studies, out of which the majority had a primarily sensory involvement with distal and symmetric distribution. The extensive biological workup did not identify a systemic disease responsible for the polyneuropathy, nor did the anamnesis reveal environmental factors or exposure to drugs or toxins that could affect the peripheral nerves. Moreover, a hereditary cause (e.g., porphyria, Charcot–Marie–Tooth disease, mitochondrial disorders) was unlikely considering the clinical presentation.

### 3.3. Blood Pressure Patterns

The blood pressure patterns and rhythm variability obtained from the 24 h ABPM are indexed in Table 5.

The diurnal and nocturnal BP values were within the normal range in our group (i.e., awake average < 135/85 mmHg, asleep average < 120/70 mmHg). However, the hyperbaric index, the area of BP excess above the upper limit of the tolerance interval, was above normal reference for both systole and diastole during the daytime and only systole during the nighttime, which would suggest an inadequate control of hypertension. The circadian rhythm of BP was also altered in our group, with 80.4% of the patients having a non-dipper profile (39.1% reduced dippers and 41.3% reverse dippers). The time-domain indices of HRV were within reference ranges, but SDNN and SDANN were closer to their lower limit (i.e., 102 ms and 92 ms, respectively).

### 3.4. Cardiovascular Dysautonomia

In the following sections, we divided the sample into two groups based on the presence or absence of CAD. We chose two phenotypes of CAD: neurogenic OH and non-dipping pattern. Notably, the mechanisms underlying the non-dipping BP profile are complex and intertwined, including perturbations of the circadian rhythm and water-sodium regulation, apart from the autonomic nervous system dysfunction [59].

Neurogenic OH. Almost one-third of the patients (31.8%) were found to have neurogenic OH. Nevertheless, 65.2% of all the subjects reported orthostatic dizziness and 15.2% had a syncope history. In our group, 21.4% (*n* = 3) of the patients with OH were asymptomatic, whereas 56.6% (*n* = 17) of the patients with orthostatic symptoms did not have OH at repeated measurements. Furthermore, we did not find an association between OH and orthostatic dizziness (*p* = 0.198). The differences between the two groups (neurogenic OH: no/yes) are presented in Table 6.

**Figure 1:**
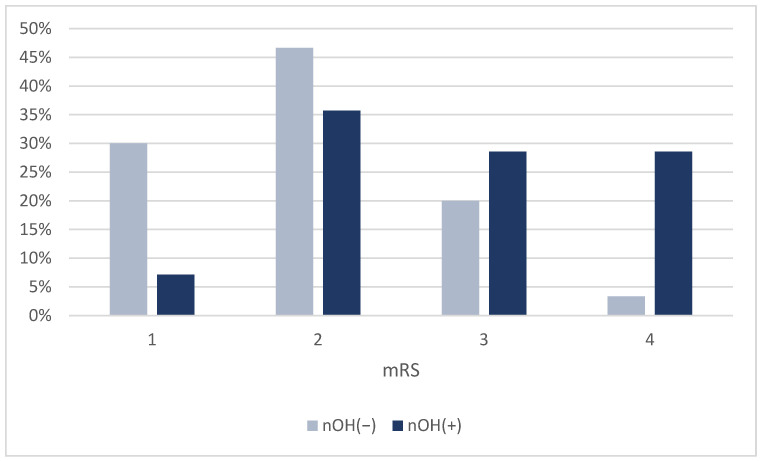
Distribution of neurogenic OH status according to the mRS.

The two groups did not differ significantly in terms of demographic factors or general PD characteristics (including levodopa-induced motor complications and motor phenotypes). All the patients with neurogenic OH were non-dippers, with significant differences from their counterparts with regard to nocturnal BP profiles (*p* = 0.016). Those with neurogenic OH reported constipation, pollakiuria, urinary urgency, and anxiety more often, whereas other individual non-motor features did not reach statistical significance between the two groups. However, the overall burden of non-motor symptoms quantified through NMSS and NMSQ was conspicuous in those with neurogenic OH as compared with their counterparts (*p* = 0.039 and 0.020, respectively); so was the disability reflected by the mRS (*p* = 0.010) (Figure 1).

After adjusting for possible confounders (Table S2. Factors associated with disability in the univariate analysis), neurogenic OH remained associated with disability (i.e., bad outcome or assistance needed: mRS > 2). Therefore, neurogenic OH could be considered a predictor of disability in PD patients with hypertension, independently of disease severity (assessed by Hoehn and Yahr stage) and duration. The multivariate analysis is presented in Table 7.

Dyslipidemia and chronic kidney disease were more frequently encountered in patients with neurogenic OH compared with the others (*p* = 0.010 and 0.032, respectively). During the nighttime, almost all BP profiles were different between the two groups, with median systolic and diastolic values, as well as hyperbaric indexes higher in the OH group. Non-dipper status was also significantly more frequent in this patient category (*p* = 0.016). The two groups were similar in terms of morning BP surge and time-domain indices of HRV. Although the time-domain indices of HRV were broadly within the reference range in the two groups, the median SDANN value was slightly reduced (the lower limit of the normal range being 92 ms).

Non-dipping profile. The differences between dippers (including normal dippers and extreme dippers) and non-dippers (including reduced dippers and reverse dippers) are illustrated in Table 8.

Patients with a non-dipper pattern of BP were significantly older than the dippers (*p* = 0.016). General PD characteristics, including motor phenotypes and levodopa-induced motor complications, did not differ between the two groups. However, some non-motor features, such as erectile dysfunction, loss of libido, and gustatory impairment, were more frequently encountered in non-dippers (*p* = 0.034, *p* = 0.020, *p* = 0.040, respectively). Univariate analysis did not find associations between age and these non-motor features; therefore, age is not expected to be a confounder. Moreover, the global non-motor burden rated on NMSS and NMSQ was also significantly higher in this group (*p* = 0.005 and 0.018, respectively). Cardiovascular comorbidities and polyneuropathy had similar distributions. Time-domain indices of HRV were slightly lower in dippers compared with non-dippers; both median SDNN and SDANN were reduced in dippers (the lower limit of the normal range being 102 ms and 92 ms, respectively). This group also had significantly higher morning BP surge (*p* = 0.001).

## 4. Discussion

We performed a pilot cross-sectional study that included patients with both sporadic PD and primary hypertension, without diabetes mellitus or other secondary causes of autonomic failure, in whom we recorded a combined 24 h ABPM and Holter ECG monitoring and measured orthostatic BP, aiming to explore the relationship between CAD and disability. Moreover, we sought associations between CAD and PD characteristics (including non-motor phenotypes), as well as cardiovascular comorbidities. In order to test the hypotheses, we assessed two phenotypes of CAD: neurogenic OH and non-dipping BP pattern (including reduced dipping and reverse dipping profiles) [56].

In our group, almost one-third of the patients (31.8%) were found to have neurogenic OH, out of which 21.4% (*n* = 3) were asymptomatic (i.e., did not complain of orthostatic dizziness and denied history of syncope; other orthostatic intolerance symptoms such as postural generalized weakness, tremulousness, fatigue/asthenia, nausea, visual blurring, tinnitus, cognitive problems, headache, neck and shoulder pain—coat hanger pain, lower back or buttocks pain, dyspnea/platypnea, palpitations or chest pain [61,62] were not addressed in this study). It is currently thought that a linear relationship between the degree of orthostatic BP fall and postural symptoms is unlikely. Palma JA et al. found that, in patients with PD and OH, orthostatic symptoms were associated with an upright mean arterial pressure < 75 mmHg [63]. Below this cutoff value, they proposed that the benefits of initiating treatment for OH exceed the risks associated with worsening SH [63]. However, this finding was not additionally endorsed. In a retrospective study enrolling 205 patients with severe OH (i.e., a SBP decrease of >60 mmHg during a head-up tilt table test), one-third were completely asymptomatic, irrespective of the origin of dysautonomia within the neuraxis (central or peripheral) [64]. In another study including 89 patients with OH identified by head-up tilt, Freeman R et al. did not find a correlation between orthostatic symptoms and the magnitude of upright SBP fall or the lowest upright SBP value, further suggesting that many patients are asymptomatic, despite prominent OH [65]. Moreover, the subgroup analysis revealed that subjects with alpha-synucleinopathies were more prone to be asymptomatic, even at lower orthostatic BP [65]. Although the mechanisms underpinning the paucity of orthostatic symptoms in OH are vastly unknown, possibilities include deficient sensory perception (due to either neurodegeneration of the structures that detect cerebral hypoperfusion, anosognosia or impaired interoception), habituation, or interindividual variations in autoregulatory mechanisms [65].

Interestingly, 65.2% of the patients in our study reported orthostatic dizziness, half of which did not have OH at repeated measurements. Moreover, in our group, we did not find an association between OH and orthostatic dizziness (*p* = 0.198). One possible explanation could be that we only tested for classic OH (sustained OH at 3 min upon standing). Apart from that, transient forms of OH have been described: initial OH with prominent BP fall (≥40 mmHg/20 mmHg or 30 mmHg/15 mmHg or 20 mmHg/10 mmHg) within 15 s of standing, which resolves within 30 s, and delayed BP recovery (SBP fall ≥ 20 mmHg), in which BP fails to recover at 30 s upon standing, but resolves within 1 min [66]. In a study comparing 173 patients with PD and 173 age—and sex—matched controls with orthostatic intolerance, Fanciulli A et al. found that transient OH occurred in 24% of the patients and 21% of the controls (*p* = 0.759); initial OH occurrence did not differ between the two groups, but delayed orthostatic BP recovery was significantly more frequent among PD patients (*p* = 0.019). This suggests that initial OH might represent an age-related phenomenon in PD, whereas delayed orthostatic BP recovery could be more intimately linked to the neurodegenerative process [66]. Delayed BP recovery, in particular, is an independent risk factor for future falls, unexplained falls, and injurious falls [67]. Furthermore, apart from the transient forms of OH that could induce orthostatic symptoms, there are other forms of sustained OH we did not record in our study: sustained OH with orthostatic BP fall ≥ 20 mmHg/10 mmHg within 1 min upon standing and delayed OH (that occurs after 3 min of standing or upright tilt); the latter is currently viewed as an early and milder form of OH that is likely to progress to OH and carries a poor prognosis [68].

Cognitive decline and OH are among the most frequent and bothersome non-motor symptoms in PD [69]. They can occur across all stages of PD and often coexist throughout its course, since their prevalence increases with aging, disease duration and disease severity [69]. However, whether their relationship is causal, associative, or bidirectional is largely unknown [69]. Various possible pathological mechanisms underlying the association between OH and cognitive impairment have been proposed: cerebral hypoperfusion driven by OH could lead to subcortical ischemic damage with subsequent cognitive decline; common neuroanatomical basis affected by PD pathology (the anterior cingulate cortex is involved in both autonomic control and cognitive tasks such as attention and motivation); shared biochemical deficits (norepinephrine acts both centrally—by modulating attention and working memory, and peripherally—by inducing hemodynamic changes upon standing up) [69]. In our study, almost half of the patients (57.4%) had cognitive decline, out of which only 3.7% had dementia, the others being classified as having MCI. The median MoCA score was 24. Notably, there were no significant differences between subjects with and without neurogenic OH with regard to cognitive decline (yes/no, *p* = 0.256), degree of cognitive impairment (*p* = 0.261), or MoCA score (*p* = 0.081). This finding comes as a surprise considering previous studies that prove the opposite. In a recent study including 226 PD patients, Longardner et al. found worse cognition among PD patients with OH in the cross-sectional analysis, and worse cognitive decline in the longitudinal analysis (which included 62 patients with a mean follow-up of 5.3 years) [70]. However, they did not assess cerebrovascular disease and hypertension (+/−hypertensive angiopathy), which are serious confounders given their relationship with cognitive impairment [71,72]. In another study enrolling 87 PD patients, out of which 30% had hypertension, Kim JS et al. found more severe cognitive impairment among those with OH [73]. In contrast, Centi J et al. found no differences regarding cognitive deficits between normotensive PD patients with and without OH while supine, with transient decrements in cognition following upright posture not attributed to a failure of cerebral autoregulation [73]. In our study, the majority of patients performed the neuropsychological tests in the seated position, so those with OH would have been expected to perform worse. Nevertheless, the comorbid hypertension in all the patients and carotid/vertebral atherosclerosis in three-quarters of the sample could account for the differences in our results.

In our study, both CAD phenotypes (i.e., neurogenic OH and non-dipping profile) were associated with an increased overall non-motor symptom burden (frequency and severity), as reflected by the NMSS and NMSQ. This is an interesting finding, since both NMSS and NMSQ include only 2 questions (out of 30 in both cases) related to OH (i.e., experiencing light-headedness, dizziness, or weakness on standing from sitting or lying position and falling due to fainting or blacking out) [47,48]. Meang Y et al. also found significantly higher mean NMSS scores in PD patients with OH as compared with those without OH (42.25 vs. 31.62, *p* < 0.001) [74]. Notably, we did not find any reported association between the non-dipping status and NMSS in the literature. Furthermore, in our group of patients, each CAD phenotype was associated with various single non-motor symptoms (revealed mainly by direct questioning rather than spontaneously offered complaints). Subjects with OH were more prone to constipation (*p* = 0.049), pollakiuria (*p* = 0.002) and urinary urgencies (*p* = 0.005), whereas non-dippers reported loss of libido (*p* = 0.020) and erectile dysfunction (*p* = 0.034) more often, which could merely reflect the spread of BP pathology within the autonomic nervous system. Considering the high burden of autonomic dysfunction (with combined symptoms pertaining to different components of the autonomic nervous system), it could also be indicative of a more prevalent body-first PD phenotype in our group of patients, as previously suggested by Horsager J et al. in a recent review [75]. However, as they also underline, these symptoms could be multifactorial, not necessarily implying neurodegeneration or dysfunction of the autonomic nervous system [75]. Moreover, the subjective complaints are not always superimposed on their objective counterparts, so an objective measurement of all autonomic functions should be employed, whenever it is possible [75]. In our study, anxiety was more prevalent among patients with neurogenic OH (*p* = 0.032). The relationship between OH and anxiety was reviewed by Perlmuter LC et al., with different studies finding an association between these manifestations [76]. Their coexistence could also predict a noradrenergic phenotype of PD, as pointed out by Chaudhuri KR et al. in a recent review [77].

Another interesting finding in our study concerns the link between neurogenic OH and cardiovascular risk factors. For instance, patients with neurogenic OH were more likely to have dyslipidemia and chronic kidney disease as compared with non-OH subjects (*p* = 0.010 and *p* = 0.032, respectively). Dyslipidemia is a classical cardiovascular risk factor, whereas chronic kidney disease increases cardiovascular morbidity and mortality, independently from classical cardiovascular risk factors [78]. Notably, chronic kidney disease is a cause of secondary CAD, provided that creatinine clearance is <8 mL/min or the patient is on dialysis [79]. However, this was not the case in our study, since severe chronic kidney disease with kidney failure was an exclusion criterion; therefore, either a reverse causality or a non-causal association between OH and chronic kidney disease could be expected.

In the same line, we also explored the relationship between CAD and ABPM measurements with prognostic value (target organ damage related to hypertension and subsequent cardiovascular risk). Patients with neurogenic OH had significantly higher median nighttime SBP and DBP than non-OH patients, which suggests an increased risk of adverse cardiovascular outcomes [44,80,81]. Moreover, the nocturnal SBP load (or hyperbaric index), which is defined as the percentage of SBP readings during nighttime that exceed the predefined threshold value (therefore depending on both average SBP levels and distribution of BP readings), was also prominently higher in the OH group (*p* = 0.008). Since the average BP values calculated on 24 h ABPM do not reflect BP variability, BP load is considered a promising marker of cardiovascular damage [82,83]. In a recent review assessing the role of BP load, Eyal O and Ben-Dov I identified various studies that had found meaningful associations between BP load and different outcomes (1. target organ damages as measured by left-ventricular hypertrophy/left-ventricular mass index, carotid intima–media thickness, common carotid diameter, carotid–femoral pulse wave velocity, brachial–ankle pulse wave velocity, retinopathy, albumin/creatinine ratio, glomerular filtration rate, and 24 h proteinuria; and 2. cardiovascular outcomes, also taking into consideration cardiovascular mortality); nevertheless, the authors acknowledge the limitations of the included studies: the lack of uniformity, the small sample size, and the exclusion of patients with significant comorbidities [84]. Unsurprisingly, in our study, subjects with neurogenic OH also exhibited more often a non-dipping pattern (*p* = 0.016); actually, all of them were either reduced dippers or reverse dippers. Apart from being a marker of CAD [59,85], the non-dipping pattern predicts all cardiovascular end-points [86,87,88]. In PD patients, Di Stefano et al. found that reverse dipping and nocturnal hypertension were associated with higher left-ventricular mass and increased prevalence of left-ventricular hypertrophy [89], which would suggest that the extrapolation of the aforementioned results to PD patients could be appropriate, but further studies are required to prove this hypothesis.

In our study, patients with neurogenic OH were significantly more disabled (with disability measured on the mRS) than those without OH (*p* = 0.010). Although the mRS was originally designed to quantify disability in patients who had suffered a stroke [90], Simuni T et al. proved its potential to be a global measure of disability in early PD, emphasizing the need for longitudinal data in order to establish its sensitivity to change over time and performance in advanced PD [91]. Later on, Taghizadeh G et al. showed that the mRS had an adequate validity and reliability to quantify the degree of disability in both on- and off-medication phases of PD patients [92]. As Weisscher N et al. previously attempted in stroke patients [93], Simuni T et al. dichotomized the scale (i.e., ≤2 versus >2) in PD patients and found significant differences between the two groups on all individual disability domain measures, thusly differentiating between a good (i.e., the ability to perform complex activities of daily living) and a bad outcome. In our study, we employed the same dichotomy; in the multivariate analysis, after adjusting for possible confounders (i.e., Hoehn and Yahr stage, PD duration, LEDD and MoCA score), neurogenic OH remained significantly associated with greater disability (*p* = 0.022). Therefore, neurogenic OH could be regarded as an independent predictor of disability in patients with PD and hypertension.

To our knowledge, this is the first study to explore the relationship between primary hypertension (assessed by 24 h ABPM) and neurogenic OH in PD patients without (other) secondary causes of autonomic dysfunction (including diabetes mellitus). Type 2 diabetes mellitus, in particular, is known for impairing cardiac autonomic markers compared with PD and hypertension, despite having lower BP levels and arterial stiffness [94]. Another strength of our study is the assessment of neurogenic OH, which implies an increase in heart rate of <15 bpm by 3 min of standing up (notably, half of our patients were on β-blockers), as opposed to most studies that did not make a distinction between neurogenic OH and non-neurogenic OH. Considering their different mechanisms, therapeutic strategies and prognostic value, it is important to distinguish between them in patients with PD. The current study presents several limitations, such as the small sample size, which might affect data precision and limit the generalizability of the results, the cross-sectional design, the absence of a control group that precludes a causal statement between the associations we found, and lastly, the incomplete data (missing values in some variables) which might secondarily affect precision. Nonetheless, we feel that the results generated in our study could lead to new hypotheses and future directions that could warrant study in PD patients with hypertension. Larger longitudinal studies focusing on patients with hypertension and PD-related neurogenic OH are required in order to better understand their interactions and to provide therapeutic solutions.

## Figures and Tables

**Table 1:** Inclusion criteria.

Established diagnosis of sporadic PD (clinically established PD according to the MDS Clinical Diagnostic Criteria for Parkinson’s disease, 2015) [13]	AND	Diagnosis of hypertension (prior history of persistent elevation in office systolic BP ≥ 140 mmHg and/or diastolic BP ≥ 90 mmHg) [41,42]; both controlled and uncontrolled hypertension were considered

**Table 2:** Exclusion criteria.

Alternative causes for parkinsonism more plausible than PD	Atypical parkinsonism Vascular parkinsonism Drug-induced parkinsonism
Secondary causes of autonomic neuropathy	Diabetes mellitus Amyloidosis Toxicity (e.g., chemotherapy, heavy alcohol consumption, and heavy metal poisoning) Systemic autoimmune inflammatory disorders (e.g., Sjogren’s syndrome) Active hematological malignancies Solid malignancies Systemic infections (i.e., HIV, chronic hepatitis B/C, Lyme disease, and syphilis) Nutritional deficiency (e.g., vitamin B12 deficiency) Dysthyroidism Liver and kidney failure
Other conditions that could interfere with cardiovascular autonomic tests	Severe anemia Deconditioning Dehydration Moderate–severe cardiac failure Artificial cardiac pacemaker Sick sinus syndrome Other major conduction disturbances
Secondary hypertension	

**Table 3:** Cardiovascular comorbidities and vasoactive drugs intake.

Cardiovascular Comorbidities	
Hypertension	
Grade 1 *n* (*%*)	11 (23.4%)
Grade 2 *n* (*%*)	14 (29.8%)
Grade 3 *n* (*%*)	22 (46.8%)
Dyslipidemia *n* (*%*)	30 (63.8%)
Atrial fibrillation *n* (*%*)	3 (6.4%)
Ischemic heart disease *n* (*%*)	4 (8.5%)
Stroke *n* (*%*)	4 (8.5%)
Carotid and/or vertebral atherosclerosis *n* (*%*)	33 (75%)
Chronic kidney disease *n* (*%*)	23 (48.9%)
Vasoactive drugs	
ACEIs *n* (*%*)	17 (36.2%)
ARBs *n* (*%*)	10 (21.3%)
CCBs *n* (*%*)	11 (23.4%)
Diuretics *n* (*%*)	11 (23.4%)
B-blockers *n* (*%*)	25 (53.2%)
Fludrocortisone *n* (*%*)	1 (2.1%)
Midodrine *n* (*%*)	3 (6.4%)
SSRIs *n* (*%*)	5 (10.6%)
SNRIs *n* (*%*)	3 (6.4%)
Quetiapine *n* (*%*)	4 (8.5%)
Clozapine *n* (*%*)	2 (4.3%)
Benzodiazepines *n* (*%*)	9 (19.2%)
Nonbenzodiazepine hypnotics *n* (*%*)	4 (8.5%)
Tamsulosin *n* (*%*)	2 (4.3%)
Mirabegron *n* (*%*)	1 (2.1%)
Antimuscarinics *n* (*%*)	3 (6.4%)
Bronchodilators *n* (*%*)	0 (0%)
Galantamine *n* (*%*)	0 (0%)
Rivastigmine *n* (*%*)	8 (17%)
Donepezil *n* (*%*)	1 (2.1%)

ACEIs = angiotensin-converting enzyme inhibitors, ARBs = angiotensin II receptor blockers, CCBs = calcium channel blockers, SSRIs = selective serotonin reuptake inhibitor, SNRIs = serotonin–norepinephrine reuptake inhibitor.

**Table 4:** PD characteristics.

Hoehn and Yahr Stage *n* (%)	
Stage 1	2 (4.3%)
Stage 2	17 (36.2%)
Stage 3	14 (29.8%)
Stage 4	12 (25.5%)
Stage 5	2 (4.3%)
PD duration, years (median, range)	9 (0–30)
MDS-UPDRS Part III (median, range)	40 (15–79)
Motor phenotype *n* (%)	
Tremor dominant	13 (27.7%)
Akinetic-rigid	8 (17%)
Mixed subtype	26 (55.3%)
Freezing of gait *n* (%)	13 (27.7%)
Gait festination *n* (%)	10 (21.3%)
Motor complications *n* (%)	
Wearing off	21 (47.7%)
Delayed on	15 (34.1%)
Unpredictable off	4 (9.3%)
No on	5 (11.4%)
Morning/night akinesia	11 (25%)
Biphasic dyskinesia	2 (4.5%)
Peak-dose dyskinesia	23 (51.1%)
Non-motor phenotype *n* (%)	
Cardiovascular domain	
Syncope	7 (15.2%)
Orthostatic dizziness	30 (65.2%)
Sleep/fatigue	
RBD	27 (58.7%)
Insomnia	29 (61.7%)
Excessive daytime sleepiness	20 (44.4%)
RLS/PLM	8 (18.7%)
Fatigue	17 (37.8%)
Mood/cognition	
Depression/Apathy	21 (44.7%)
Anxiety	23 (48.9%)
Neurocognitive disorder	27 (57.4%)
Perceptual problems	
Hallucinations	15 (31.9%)
Diplopia	11 (24.4%)
Gastrointestinal tract	
Drooling	15 (33.3%)
Dysphagia	17 (37.8%)
Nausea	8 (17.8%)
Early satiety	10 (22.2%)
Bloating	4 (8.9%)
Constipation	31 (67.4%)
Urinary	
Urinary urgency	29 (63%)
Pollakiuria	24 (52.2%)
Nocturia	31 (67.4%)
Urinary retention	13 (28.3%)
Sexual function	
Erectile dysfunction	9 (40.9%)
Anejaculation	1 (7.1%)
Loss of libido	10 (45.5%)
Thermoregulation	
Hyperhidrosis	22 (50%)
Night sweats	16 (36.4%)
Sensory and special senses	
Pain/paresthesia	10 (22.2%)
Olfactory dysfunction	20 (43.5%)
Gustatory dysfunction	8 (17.8%)
Blurred vision	10 (22.2%)
Miscellaneous	
Seborrhea	2 (4.4%)
Weight loss	9 (20%)
Weight gain	1 (2.2%)
Edema	10 (22.2%)
Impulse control disorders	6 (12.8%)
MoCA (median, range)	24 (11–30)
NMSS (median, range)	67 (0–213)
NMSQ (median, range)	15 (0–76)
COMPASS 31 (median, range)	17 (1–67)
SCOPA-AUT (median, range)	21 (0–42)
Disability (median, range)	
IADL	6 (0–8)
ADL	8 (0–10)
Barthel Index	75 (15–100)
mRS	2 (1–4)
LEDD, mg (median, range)	920 (188–2269)
Antiparkinsonian drugs	
Oral levodopa *n* (%)	43 (91.5%)
Immediate release *n* (%)	40 (85.1%)
Controlled release *n* (%)	11 (23.4%)
Scheduled dosing times (median)	4
Dopamine agonists *n* (%)	27 (57.4%)
Oral *n* (%)	17 (36.2%)
Transdermal *n* (%)	10 (21.3%)
MAO-B inhibitors *n* (%)	21 (44.7%)
Oral COMT inhibitors *n* (%)	16 (34%)
NMDA receptor antagonists *n* (%)	11 (23.4%)
Device-aided therapy *n* (%)	12 (25.5%)
Polyneuropathy *n* (%)	15 (34.9%)

RLS = Restless Legs Syndrome, PLM = Periodic Leg Movement, MAO-B = type-B monoamine oxidase, COMT = cathecol-O-methyltransferase, NMDA = N-methyl-D-aspartate.

**Table 5:** BP patterns on 24 h ABPM.

Daytime	
SBP, mmHg (median, range)	120 (102–145)
DBP, mmHg (median, range)	70 (60–111)
HIS, mmHg.h (median, range)	11 (0–152)
HID, mmHg.h (median, range)	9 (0–83)
Nighttime	
SBP, mmHg (median, range)	116.5 (72–156)
DBP, mmHg (median, range)	66 (47–93)
HIS, mmHg.h (median, range)	17 (0–264)
HID, mmHg.h (median, range)	0 (0–67)
Dipper profile	
Extreme dipper *n* (*%*)	1 (2.2%)
Normal dipper *n* (*%*)	8 (17.4%)
Reduced dipper *n* (*%*)	18 (39.1%)
Reverse dipper *n* (*%*)	19 (41.3%)
Morning surge, mmHg (median, range)	10 (−48; +64)
Time-domain indices of HRV	
SDNN, ms (median, range)	108 (35–249)
SDANN, ms (median, range)	92 (31–190)
RMSSD, ms (median, range)	29 (8–312)

HIS = hyperbaric impact systole, HID = hyperbaric impact diastole, SDNN = standard deviation of normal-to-normal (NN) intervals, SDANN = standard deviation of the average NN intervals, RMSSD = root-mean-square of successive differences.

**Table 6:** Differences between patients with and without neurogenic OH.

	Neurogenic OH (−) *n* (*%*)	Neurogenic OH (+) *n* (*%*)	*p*-Value
	30 (68.2%)	14 (31.8%)	
Demographic factors			
Age, years (median, range)	69 (48–78)	71 (60–83)	0.318
Sex, males *n* (*%*)	18 (40.91%)	8 (18.18%)	0.858
PD characteristics			
Hoehn and Yahr stage (median, range)	3 (1–5)	3 (2–5)	0.735
PD duration, years (median, range)	9 (0–30)	7.5 (0–24)	0.677
MDS-UPDRS Part III (median, range)	45 (15–79)	40 (29–66)	0.92
LEDD, mg (median, range)	1064.5 (188–2170)	889 (188–2269)	0.45
Device-aided therapy *n* (*%*)	10 (22.73%)	2 (4.55%)	0.186
mRS (median, range)	2 (1–4)	3 (1–4)	0.01
Neurocognitive disorder *n* (*%*)	16 (36.36%)	10 (22.73%)	0.256
Non-motor phenotype *			
Constipation *n* (*%*)	16 (37.21%)	12 (27.91%)	0.049
Pollakiuria *n* (*%*)	10 (23.26%)	12 (27.91%)	0.002
Urinary urgency *n* (*%*)	14 (32.56%)	13 (30.23%)	0.005
Anxiety *n* (*%*)	11 (25%)	10 (22.73%)	0.032
NMSS (median, range)	59 (0–157)	87 (13–213)	0.039
NMSQ (median, range)	13.5 (0–72)	20 (8–76)	0.02
Cardiovascular comorbidities			
Dyslipidemia *n* (*%*)	16 (36.36%)	13 (29.55%)	0.01
Atrial fibrillation *n* (*%*)	1 (2.27%)	2 (4.55%)	0.179
Ischemic heart disease *n* (*%*)	1 (2.27%)	2 (4.55%)	0.179
Stroke *n* (*%*)	2 (4.55%)	1 (2.27%)	0.953
Carotid and/or vertebral atherosclerosis *n* (*%*)	19 (46.34%)	12 (29.27%)	0.278
Chronic kidney disease *n* (*%*)	11 (25%)	10 (22.73%)	0.032
24-h ABPM measurements			
Daytime			
SBP, mmHg (median, range)	121 (102–136)	120 (109–145)	0.561
DBP, mmHg (median, range)	69.5 (60–111)	72 (65–88)	0.154
HIS, mmHg.h (median, range)	14 (0–102)	18.5 (0–152)	0.478
HID, mmHg.h (median, range)	7.5 (0–56)	14.5 (0–83)	0.154
Nighttime			
SBP, mmHg (median, range)	112.5 (72–153)	129 (111–156)	0.001
DBP, mmHg (median, range)	64.5 (47–93)	77 (53–87)	0.007
HIS, mmHg.h (median, range)	16 (0–264)	84 (4–255)	0.008
HID, mmHg.h (median, range)	0 (0–30)	4 (0–67)	0.108
Dipper profile			0.016
Extreme dipper *n* (*%*)	1 (2.33%)	0 (0%)	
Normal dipper *n* (*%*)	8 (18.6%)	0 (0%)	
Reduced dipper *n* (*%*)	13 (30.23%)	3 (6.98%)	
Reverse dipper *n* (*%*)	8 (18.6%)	10 (23.26%)	
Time-domain indices of HRV			
SDNN, ms (median, range)	107.5 (53–199)	103 (35–249)	0.705
SDANN, ms (median, range)	91.5 (47–190)	89.5 (31–168)	0.579
RMSSD, ms (median, range)	31 (10–214)	18.5 (8–312)	0.545
Morning surge, mmHg (median, range)	11.5 (−48; +64)	10 (−16; +37)	0.832
Polyneuropathy *n* (*%*)	7 (17.07%)	6 (14.63%)	0.176

HIS = hyperbaric impact systole, HID = hyperbaric impact diastole, SDNN = standard deviation of normal-to-normal (NN) intervals, SDANN = standard deviation of the average NN intervals, RMSSD = root-mean-square of successive differences; * only the phenotypes with statistically significant results.

**Table 7:** Neurogenic OH as a predictor of disability, adjusted for Hoehn and Yahr stage, PD duration, LEDD, and MoCA score (logistic regression).

Variables	B	OR	95% C.I. for OR		***p*** **-Value**
			Lower	Upper	
Neurogenic OH	−2.430	0.088	0.011	0.710	0.022
Hoehn and Yahr stage	1.309	3.702	0.867	15.805	0.077
PD duration	0.051	1.052	0.872	1.268	0.596
LEDD	0.001	1.001	0.998	1.003	0.686
MoCA score	−0.061	0.941	0.764	1.159	0.569

**Table 8:** Differences between dippers and non-dippers.

	Dipper Profile *n* (*%*)	Non-Dipper Profile *n* (*%*)	*p*-Value
	9 (19.1%)	37 (78.7%)	
Demographic factors			
Age, years (median, range)	60 (51–75)	72 (48–83)	0.016
Sex, males *n* (*%*)	6 (12.77%)	21 (44.68%)	0.434
PD characteristics			
Hoehn and Yahr stage (median, range)	3 (1–4)	3 (1–5)	0.469
PD duration (median, range)	9 (1–30)	8 (0–24)	0.978
MDS-UPDRS Part III (median, range)	48 (20–73)	40 (15–79)	0.658
LEDD (median, range)	1190 (268–2070)	875 (188–2269)	0.353
Device-aided therapy *n* (*%*)	4 (8.51%)	8 (17.02%)	0.311
mRS (median, range)	2 (1–3)	2 (1–4)	0.123
Neurocognitive disorder *n* (*%*)	4 (8.51%)	23 (48.94%)	0.315
Non-motor phenotype *			
Erectile dysfunction *n* (*%*)	0 (0%)	9 (40.91%)	0.034
Loss of libido *n* (*%*)	0 (0%)	10 (45.45%)	0.02
Gustatory dysfunction *n* (*%*)	0 (0%)	7 (15.56%)	0.04
NMSS (median, range)	15 (0–95)	77.5 (9–213)	0.005
NMSQ (median, range)	7.5 (0–72)	16 (6–76)	0.018
Cardiovascular comorbidities			
Dyslipidemia *n* (*%*)	3 (6.38%)	26 (55.32%)	0.088
Atrial fibrillation *n* (*%*)	1 (2.13%)	2 (4.26%)	0.793
Ischemic heart disease *n* (*%*)	0 (0%)	4 (8.51%)	0.554
Stroke *n* (*%*)	0 (0%)	4 (8.51%)	0.554
Carotid and/or vertebral atherosclerosis *n* (*%*)	5 (11.36%)	28 (63.64%)	0.202
Chronic kidney disease *n* (*%*)	3 (6.38%)	19 (40.43%)	0.367
24-h ABPM measurements			
Time-domain indices of HRV			
SDNN, ms (median, range)	93 (87–167)	108 (35–249)	0.792
SDANN, ms (median, range)	87 (62–133)	93 (31–190)	0.901
RMSSD, ms (median, range)	17 (10–214)	30 (8–312)	0.203
Morning surge, mmHg (median, range)	22 (+10; +64)	8 (−48; +37)	0.001
Polyneuropathy *n* (*%*)	2 (4.65%)	13 (30.23%)	0.48

SDNN = standard deviation of normal-to-normal (NN) intervals, SDANN = standard deviation of the average NN intervals, RMSSD = root-mean-square of successive differences; * only the phenotypes with statistically significant results.

## Data Availability

Data are contained within the article.

## Supplementary Materials

The following supporting information can be downloaded at: https://www.mdpi.com/article/10.3390/jcm14072225/s1, Table S1. Patient’s medical chart, Table S2. Factors associated with disability in the univariate analysis.
