# Supplementary material for: Cardiovascular Dysautonomia in Patients with Parkinson’s Disease and Hypertension: A Cross-Sectional Pilot Study
Source: J Clin Med. 2025 Mar 25;14(7):2225. doi: 10.3390/jcm14072225 (PMC11989698; doi:10.3390/jcm14072225)
Supplement: Supplementary file 1 [file jcm-14-02225-s001.zip › Table S1.pdf]

Table S1. Patient's medical chart.

Pacient code:
Date:

- **Demographics/socio-cultural context**
  - Age (years)
  - Sex (female/male)
  - Living environment (rural/urban)
  - Work status (employed/retired)
  - Occupational toxic exposure (yes/no)
  - Alcohol consumption (yes/no), smoker (yes/no)

- **Relevant comorbidities (cardiovascular risk factors) (yes/no)**
  - Ischemic heart disease
  - History of stroke
  - Atherosclerosis (and territory involved)
  - Dyslipidemia
  - Chronic kidney disease

- **Arterial hypertension (staging)**

- **Vasoactive drugs (yes/no)**
  - Antihypertensive drugs (ACEIs, ARBs, CCBs, diuretics, β-blockers)
  - Antihypotensive drugs (fludrocortisone, midodrine)
  - Psychoactive drugs (antidepressants/hypnotic drugs/antipsychotics: SSRIs, SNRIs, clozapine, quetiapine, benzodiazepines, nonbenzodiazepine hypnotics)
  - A1-blockers (doxazosin, tamsulosin)
  - B3-adrenergic agonists (mirabegron)
  - B2-adrenergic agonists (albuterol, formoterol)
  - Anticholinergic drugs (oxybutynin, solifenacin, trospium chloride, trihexyphenidyl, benztropine)
  - Acetylcholinesterase inhibitors (galantamine, rivastigmine, donepezil, pyridostigmine, neostigmine)

- **PD – clinical evaluation (with motor fluctuations journal and related scales)**
  - Disease duration (from motor onset)
  - Motor symptoms
    - Hoehn and Yahr grading scale (in OFF)
    - MDS-UPDRS Part III (in overnight OFF)
    - Motor complications (wearing off, delayed on, unpredictable off, no on, morning/night

akinesia, biphasic dyskinesia, peak-dose dyskinesia)
- Current motor symptoms (including freezing and festination of gait)
- Motor phenotype (tremor dominant, akinetic-rigid, mixed subtype)

- Non-motor symptoms
    - Scales (NMSS, NMSQ, COMPASS 31, SCOPA-AUT)
    - Neuropsychiatric symptoms (depression/apathy, anxiety, hallucinations, ideation, cognitive impairment – MoCA)
    - Sleep disorders (RLS/PLM, RBD, sleep induction/maintenance/mixed insomnia, excessive daytime sleepiness)
    - Bladder dysfunction (pollakiuria, urgency, nocturia, urinary retention)
    - Sexual dysfunction (erectile disfunction, loss of libido, anejaculation)
    - OH (orthostatic dizziness, syncope, orthostatic intolerance)
    - Thermoregulatory dysfunction (hyper/hypohidrosis, night sweats)
    - Gastrointestinal dysfunction (drooling/sialorrhea, xerostomia, gastrointestinal reflux, nausea and vomiting, early satiety, bloating, constipation, bowel incontinence)
    - Sensory symptoms (pain, paresthesia, olfactory dysfunction, gustatory dysfunction)
    - Others (Mental/physical fatigue, diplopia, blurry vision, seborrhea, weight gain, weight loss, peripheral edema)
- Disability scales (IADL, ADL, Barthel Index, mRS)
- Medication
    - Levodopa (immediate release/controlled release, number of doses)
    - Dopamine agonists (oral/transdermal)
    - MAO-B inhibitors
    - COMT inhibitors
    - NMDA receptor antagonists
    - Device-assisted therapy
    - LEDD

- **Paraclinical workup**
    - Blood tests: hemoglobin A1c, thyroid stimulating hormone, vitamin B12 levels, lipid profile, uric acid, electrophoresis with immunofixation, creatinine, BUN, complete blood count, brain natriuretic peptide, erythrocyte sedimentation rate, C reactive protein, HIV antibodies, hepatitis B surface antigen, hepatitis C antibodies
    - 24 h ABPM (coupled with daytime and sleep journal): average diurnal, nocturnal and 24 hour SBP and DBP, dipping profile (normal, extreme, reduced, reversed dipping), morning surge, BP load
    - 24 h ECG Holter: HRV (frequency and time domain), brady/tachyarrhythmias

- Carotid and transcranial Doppler ultrasound: carotid intima-media thickness, atherosclerotic plaques (number, echogenicity, location, thickness, area), carotid flow velocity<br>- Nerve conduction studies: sensory and motor velocities, compound sensory and muscle action potentials (SNAPs and CMAPs)<br>- ECG: Rhythm, heart axis, heart rate, P wave, PR segment, QRS complex, R wave, QT segment<br>- OH test: 1,3,5 minutes.

ACEIs = angiotensin-converting enzyme inhibitors, ARBs = angiotensin II receptor blockers, CCBs = calcium channel blockers, SSRIs = selective serotonin reuptake inhibitor, SNRIs = serotonin-norepinephrine reuptake inhibitor, PD = Parkinson's disease, MDS-UPDRS = Movement Disorder Society-sponsored revision of the Unified Parkinson's Disease Rating Scale, NMSS = Non-Motor Symptoms Scale for Parkinson's Disease, NMSQ = Non-Motor Symptoms Questionnaire, COMPASS 31 = Composite Autonomic Symptom Score-31, SCOPA-AUT = Scales for Outcomes in Parkinson's Disease-Autonomic Dysfunction, MoCA = Montreal Cognitive Assessment, RLS = Restless Legs Syndrome, PLM = Periodic Leg Movement, RBD = Rapid Eye Movement Sleep Behaviour Disorder, OH = orthostatic hypotension, IADL = Lawton Instrumental Activities of Daily Living, ADL = Activities of Daily Living, Barthel Index = Barthel Index for Activities of Daily Living, mRS = modified Rankin scale, MAO-B = type-B monoamine oxidase, COMT = cathecol-O-methyltransferase, NMDA = N-methyl-D-aspartate, LEDD = levodopa equivalent daily dose, BUN = blood urea nitrogen, ABPM = ambulatory blood pressure monitoring, SBP = systolic blood pressure, DBP = diastolic blood pressure, BP = blood pressure, HRV = heart rate variability, SNAPs = sensory action potentials, CMAPs = compound muscle action potentials, ECG = electrocardiogram.
