# Supplementary material for: Cardiovascular Dysautonomia in Patients with Parkinson’s Disease and Hypertension: A Cross-Sectional Pilot Study
Source: J Clin Med. 2025 Mar 25;14(7):2225. doi: 10.3390/jcm14072225 (PMC11989698; doi:10.3390/jcm14072225)
Supplement: Supplementary file 1 [file jcm-14-02225-s001.zip › Table S2.pdf]

**Table S2.** Factors associated with disability in the univariate analysis.

| | Disability (-) *N (%)* 31 (66%) | Disability (+) *N (%)* 16 (34%) | ***p*-Value** |
|---|---|---|---|
| **Neurogenic OH** *N (%)* | 6 (42.9%) | 8 (57.1%) | 0.028 |
| **Hoehn and Yahr stage** *(median)* | 2 | 4 | 0.001 |
| **PD duration** *(years, median)* | 7 | 12 | 0.026 |
| **LEDD** *(mg, median)* | 843 | 1220 | 0.021 |
| **MoCA score** *(median)* | 26 | 22 | 0.025 |
| **MDS-UPDRS Part III** *(median)* | 40 | 45 | 0.271 |
| **Age** *(years, median)* | 69 | 71 | 0.345 |
| **PD subtype** *N* | | | 0.207 |
| • **tremor dominant** | 7 | 6 | |
| • **rigid-akinetic** | 4 | 4 | |
| • **mixt** | 20 | 6 | |

OH = orthostatic hypotension, PD = Parkinson's disease, LEDD = levodopa equivalent daily dose, MoCA = Montreal Cognitive Assessment, MDS-UPDRS = Movement Disorder Society-sponsored revision of the Unified Parkinson's Disease Rating Scale.
